# Tumor microenvironment enriches the stemness features: the architectural event of therapy resistance and metastasis

**DOI:** 10.1186/s12943-022-01682-x

**Published:** 2022-12-22

**Authors:** Palanisamy Nallasamy, Rama Krishna Nimmakayala, Seema Parte, Abhirup C. Are, Surinder K. Batra, Moorthy P. Ponnusamy

**Affiliations:** 1 Department of Biochemistry and Molecular Biology, University of Nebraska Medical Center, Omaha, NE 68198-5870 USA; 2 Fred and Pamela Buffett Cancer Center, University of Nebraska Medical Center, Omaha, NE USA; 3 Eppley Institute for Research in Cancer and Allied Diseases, University of Nebraska Medical Center, Omaha, NE USA

**Keywords:** Tumor microenvironment, Cancer stem cells, Fibroblasts, Metastatic stem cells, Therapy resistance

## Abstract

Cancer divergence has many facets other than being considered a genetic term. It is a tremendous challenge to understand the metastasis and therapy response in cancer biology; however, it postulates the opportunity to explore the possible mechanism in the surrounding tumor environment. Most deadly solid malignancies are distinctly characterized by their tumor microenvironment (TME). TME consists of stromal components such as immune, inflammatory, endothelial, adipocytes, and fibroblast cells. Cancer stem cells (CSCs) or cancer stem-like cells are a small sub-set of the population within cancer cells believed to be a responsible player in the self-renewal, metastasis, and therapy response of cancer cells. The correlation between TME and CSCs remains an enigma in understanding the events of metastasis and therapy resistance in cancer biology. Recent evidence suggests that TME dictates the CSCs maintenance to arbitrate cancer progression and metastasis. The immune, inflammatory, endothelial, adipocyte, and fibroblast cells in the TME release growth factors, cytokines, chemokines, microRNAs, and exosomes that provide cues for the gain and maintenance of CSC features. These intricate cross-talks are fueled to evolve into aggressive, invasive, migratory phenotypes for cancer development. In this review, we have abridged the recent developments in the role of the TME factors in CSC maintenance and how these events influence the transition of tumor progression to further translate into metastasis and therapy resistance in cancer.

## Introduction

Cancer development is a multifaceted process in which the somatic cells attain and accrue numerous genetic and epigenetic changes resulting from uncontrolled cell growth, aggressive replication, and constant mutation leading to the progression of the disease [1]. A niche surrounding in tumor consists of complex tissues organized into discrete cell types originating from the nearby mesenchymal stroma. This mesenchymal stroma forms a favorable *milieu* around the tumor cells, which is denoted as the “tumor microenvironment (TME)” [2]. Mesenchymal stroma is a multi-cellular ecosystem consisting of stromal, infiltrating immune, inflammatory, endothelial, adipocyte, and fibroblast cells in the TME. These discrete cell types establish the TME to facilitate and sustain the hallmarks of cancer via mutual communication with cancer cells to subsidize their functional diversity. In that event, the neoplastic cells and their surrounding TME release a series of signals that translate into a pathological entity to evolve the advancement of cancer. These cross-talks lead to cancer cell plasticity, invasive and migratory potential, and the eventual growth of a full-blown tumor. In conjunction with cancer cells, this developed microenvironment is critical in the development of advanced malignancies [3].

**Fig. 1:**
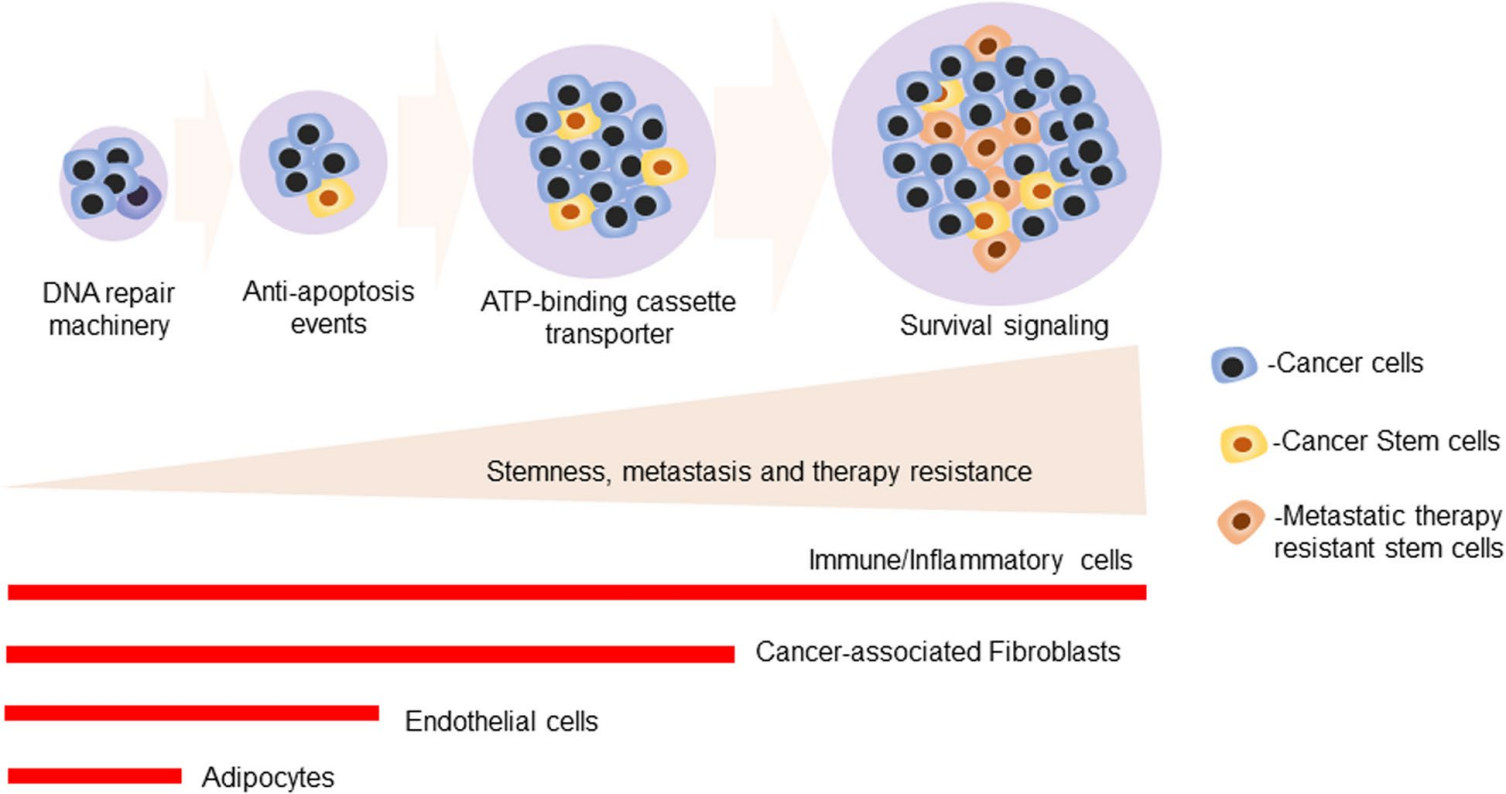
Overview of the non-genetic determinants and cascade of events that drives the stemness to initiate metastasis and therapy resistance in advanced malignancies: The schematic diagram illustrated that non-genetic determinants and arrays of cascade events played a crucial role in the development of advanced cancer with progressively increased stemness to initiate metastasis and therapy resistance

Chemotherapy is a preliminary treatment option for cancer patients [4]. The majority of the patients face a lapse in drug effectiveness due to the development of therapy resistance after post-cancer treatment. Resistance to therapy results from various biological mechanisms, including DNA repair, genetic and epigenetic modification, metabolic reprogramming, increased angiogenesis, and altered TME. Hence, treatment poses a formidable challenge to clinicians and researchers who primarily treat metastatic and malignant cases, thus necessitating the identification and implementation of target-specific therapy to develop precision medicine. Most patients succumb early during treatment due to frequent tumor relapse after the first line of treatment in many solid tumors [5]. Developments of multi-drug resistance in almost all solid malignancies are considered a major problem in onco-therapeutic treatment modalities. Besides genetic predisposition and hereditary mutations, emerging studies prove that a non-genetic component also plays a vital role in therapy resistance. In contrast, surgery, radiation, and chemo therapies cannot sufficiently address a sharp rise in cancer-related deaths worldwide. We described how non-genetic determinants and a cascade of events promoting stem cell enrichment might initiate metastasis and therapy resistance in advanced malignancies in Fig. 1.

**Fig. 2:**
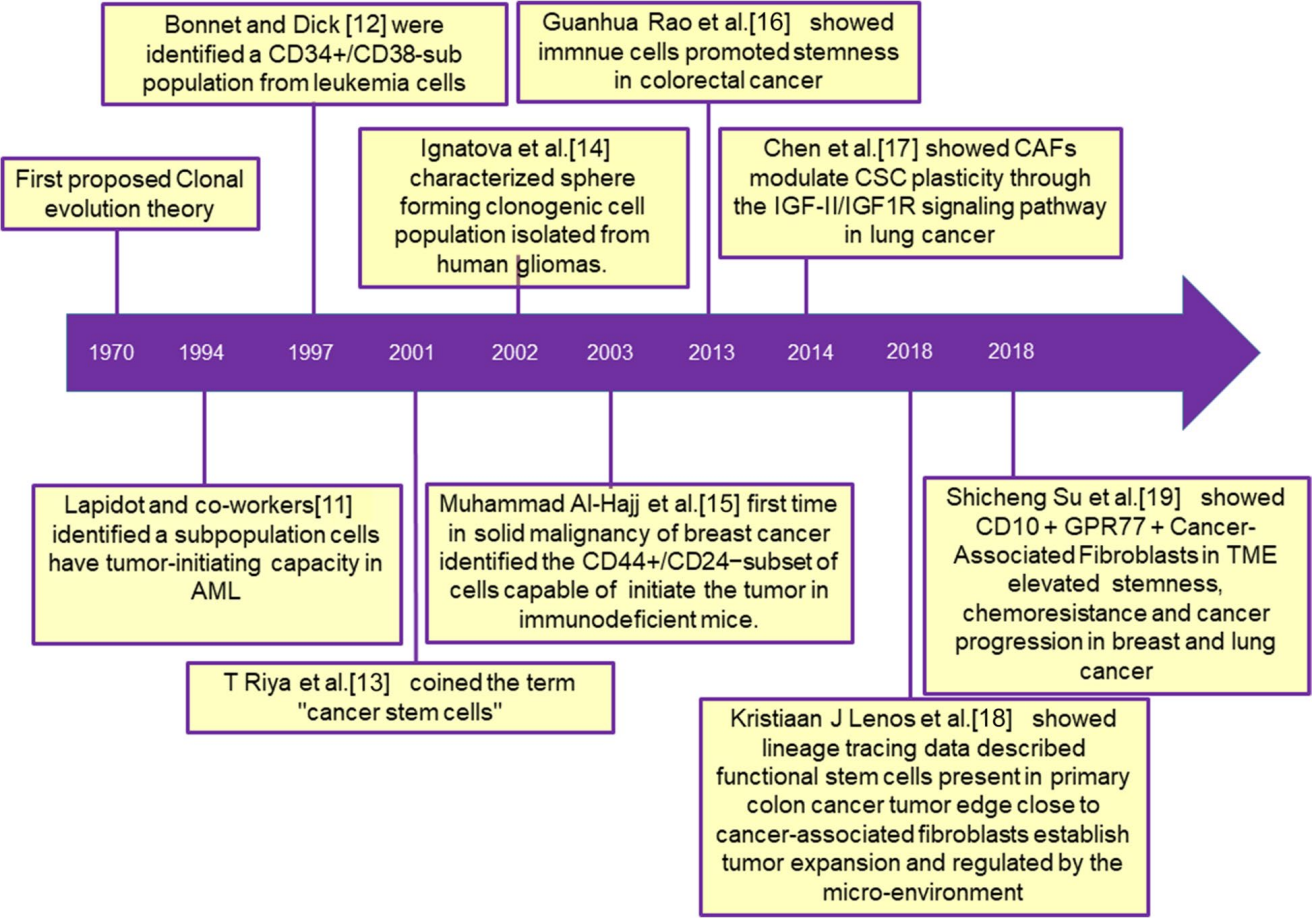
The complied chart of timeline events in the tumor microenvironment-mediated stemness research: The diagram showed the tumor microenvironment, and stemness-related research findings appeared in different periods [11–19]

Earlier high-throughput sequencing studies show that tumor progression and heterogeneity are based on the acquisition of mutations in different stages of tumor development [6]. Interestingly, studies also showed that a single clone might display functional variation in the populations [7], thus pointing towards a vital role of non-genetic determinants in shaping the differential or various functionality of subclones of cells, thus leading to differential survival outcomes in response to the treatment regimen [8]. The current treatment approaches eliminate the cancer-associated burden; however, the effect of therapies remains obsolete against the quiescent sub-population of cells that we address as cancer stem cells (CSCs) or tumor-initiating cells (TICs). The clonal evolution theory postulated in 1970 that cancer cells acquire genetic mutations during tumor evolution or transformation, resulting in the accumulation of sub-clonal populations with diverse phenotypes and heterogeneous characters. They hypothesized that a small population of cells called CSCs with distinct mutational and epigenetic profiles drives tumor initiation and progression while effluxing the drug introduced into the targeted cancer tissue, ultimately contributing to the development of therapy resistance [9]. Recent findings assume that this small subset of cancer-initiating/metastasizing populations governs the cancer progression to initiate/maintain metastasis and therapy resistance. Therefore, understanding communication between TME and residual CSC populations may be a robust network responsible for tumor relapse and recurrence, leading to the mortality of cancer patients [10]. To comprehend the ongoing research in TME and CSCs, we enlisted a timeline of events in the chronology in Fig. 2.

This review discusses the present knowledge on the TME, its involvement in CSC maintenance, and how these processes translate into metastasis and therapeutic resistance in cancer. We also assessed ongoing TME-targeted clinical studies and investigated potential therapeutic new ways to target this TME-CSC interplay in cancer patients to reduce medication resistance.

### Cancer stem cells and their unique features

Stem cell theory, proposed four decades ago, claims that a limited number of cells are responsible for developing healthy tissue and tumor growth. Many cancers have been verified to include CSCs, but their enrichment mechanism is poorly explored. Recent advancements in lineage tracing techniques provide a better understanding of the plasticity, renewal, and therapy response. In 1997, Bonnet and Dick identified a CD34^+^/CD38^−^ sub population from leukemia cells that initiated tumors in NOD/SCID mice [12]. However, later studies also demonstrated the existence of this CSCs in other solid malignancies [15]. CSCs are tumor-initiating cells present within the tumor capable of self-renewal, differentiate into diverse cell types, and cause tumorigenicity and chemo or radiotherapy resistance in all solid malignancies [20]. Recent development in cell surface marker flowcytometric or magnetic sorting techniques led to the efficient identification of CSCs in solid malignancies [21, 22]. Chemoresistance develops due to changes in the expression of ABC transporter proteins, EMT, and angiogenesis. The glycocalyx, which contains sugar moieties and glycoproteins, exists on the outer side of the plasma membrane of cancer cells and interacts with the TME to drive cancer progression by convincing cell-cell, ECM contact, cell adhesion, and stemness acquisition [23]. Importantly, altered glycosylation of stemness markers plays a crucial role progression of the disease [24]. CSCs are distinguished from other cells of the tumor mass based on their unique phenotypic characteristics and functionality, and distinct CSCs modulate metastasis by reprogrammed cellular metabolism in pancreatic cancer [25]. Intrinsic and extrinsic factors regulate CSCs maintenance, and the TME is responsible for some critical signaling and communication events leading to metastasis and therapy resistance. TME factors and cancer cell communication are vital signaling elements to maintain CSC heterogeneity and plasticity in cancer cells.

CSC-related genes and the associated signaling pathways promote an oncogenic transformation in cancer cells. A pluripotent stem cell marker, Nanog, is associated with the STAT3 molecule to activate therapy resistance genes ABCB1 and MDR1 in breast and ovarian cancer cells [26]. These molecules mediate a cross-talk between hyaluronan-CD44 signaling in cancer cells to maintain the stemness in cancer cells [27]. Recent findings showed a critical role of PI3Ks in maintaining tumorigenicity in colorectal CSCs [28]. ECM-derived factors are considered predominant molecules modulating the progression of cancer development. These ECM factors are essential in CSC cross-talk and maintaining stemness in tumor cells. Hyaluronic acid, laminin, fibronectin, and collagen are significant factors in ECM to initiate biochemical events and cross-talk with cancer cells, promoting stemness and therapy resistance in various cancers [29, 30]. EMT plays an essential role in the invasive and metastatic activity of CSCs, as reported in a recent study [31]. Further, Malanchi et al. [32] showed that the CSC population is responsible for metastatic colonization and initial expansion of cancer cells at the secondary site and confirmed that an extracellular component, periostin, was responsible for educating infiltrating tumor cells to cause metastasis. Hence, intense research is required to target the receptor-ligand interaction in stroma-CSC cross talk.

### Tumor microenvironment maintains the ecosystem in solid tumors

**Fig. 3:**
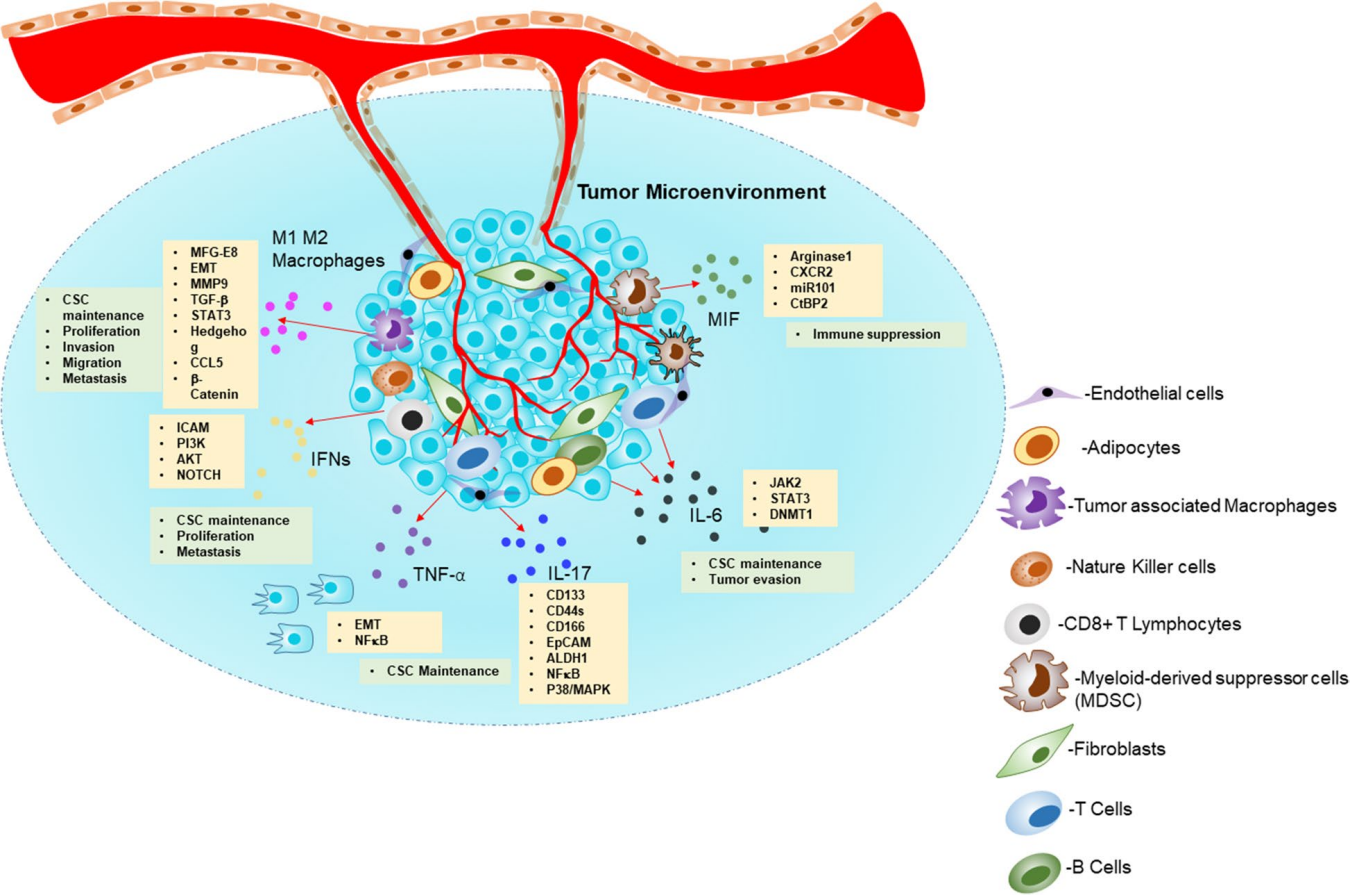
The tumor microenvironment and their cross-talk signal mechanisms in immune cells, inflammatory cells, endothelial cells, adipocytes, and fibroblasts drive the cancer stem cell maintenance: The schematic illustration summarizes the microenvironmental tumor factors secreting signaling molecules’ role in the CSC maintenance. Abbreviations: MFG-E8-Milk fat globule-EGF factor 8 protein; EMT-Epithelial-mesenchymal transition; MMP 9-Matrix metallopeptidase 9; TGF-β- Transforming growth factor-β; CCL5- C-C motif chemokine ligand 5; ICAM1-Intercellular adhesion molecule 1; IFNs-Interferons; TNF-α-Tumor necrosis factor-α; IL 6- Interleukin 6; ALDH1-Aldehyde dehydrogenase 1; DNMT1-DNA methyltransferase 1; CtBP2-C-terminal-binding protein 2 ; MIF-Macrophage migration inhibitory factor

TME encompasses various components such as fibroblasts, immune cells, endothelial cells, extracellular matrix, cytokines, growth factors, and hormones surrounding tumor cells nourished by a vascular network (Fig. 3) [33]. TME plays a pivotal role in stemness maintenance apart from tumor progression. The incessant cross-talk between cancer cells and surrounding stroma containing TME factors such as immune cells, inflammatory cells, endothelial cells, adipocytes, and fibroblasts plays a major role in microenvironment-mediated stemness enrichment [34]. We have enlisted key TME factors, and their influences on the enrichment of stemness are listed in Table 1. In forthcoming titles, we discuss the TME factor’s role in stemness maintenance and how it impacts metastasis and therapy failure in cancer patients.

**Table 1:** Key tumor microenvironmental components, stemness factors and their functions in CSCs maintenance

S.No.	TME Stemness factors	Cancer/cells	Functions	References
1.	Interleukin 8	Breast Cancer	It promoted the EMT and acquisition of stemness in MCF-7 cells	[35]
2.	Platelet-activating factor (PAF)	Ovarian cancer	It endorsed progression and chemoresistance of ovarian cancer via PAF/PAF-R inflammatory pathway.	[36]
3.	YY1	Lung cancer	It stabilized HIF-1α protein through YY1/HIF-1α axis promotes stemness in lung cancer cells	[37]
4.	Mitochondrial ribosomal protein S18-2	Mouse fibroblasts	It positively correlated with KLF4 to enhance stemness in primary mouse embryonic fibroblasts.	[38]
5.	Monoamine oxidase A	Prostate cancer	It induced the IL-6 transcription through Twist1/IL-6/STAT3 pathway	[39]
6.	Chemokine (C-C motif) ligand 8 (CCL8)	GBM	It activated the ERK1/2 phosphorylation to mediate stemness.	[40]
7.	IL-10	Non-small cell lung cancer (NSCLC)	It promoted through JAK1/STAT1/NF-κB/Notch1 signaling to maintain CSCs-like cells	[41]
8.	HGF and IL6	Hepatocellular carcinoma (HCC)	It secreted from CAFs promoted the stemness through phosphorylation of STAT3	[42]
9.	CCL2	esophageal cancer	It is derived from tumor-associated macrophages to enhances the stemness	[43]
10.	Phospholipase D2 (PLD2)	colon cancer	It induced senescence in nearby fibroblasts leads to senescence-associated secretory phenotype to promotes stemness	[44]
11.	BMP4	oral cancer	It is derived from CAFs and regulated the self-renewal.	[45]
12.	Osteopontin	colon cancer	It is derived from CAFs and drives in situ clonogenicity	[18]
13.	Exosomal lncRNA H19	colorectal cancer	It is fostered the stemness and chemoresistance to activate β- catenin pathway	[46]
14.	rhIL-8	Ovarian cancer	It promoted M2 macrophage polarization and stemness in SKOV3 cells	[47]
15.	CD10 GPR77	Breast cancer	It activated NFκB via p65 phosphorylation and acetylation to promote tumor formation and stemness.	[19]
16.	Interleukin 7-producing fibroblasts	Breast cancer	It modulated CXCL12/CXCR4 axis to enhance breast tumor cell stemness	[48]
17.	CD4 + CD25 + Tregs	Breast cancer	It activated NFκB-CCL1 signaling, to promotes the stemness in breast cancer cells.	[49]
18.	High-mobility group box 1 (HMGB1)	Breast cancer	It activated the HMGB1-TLR4 axis to maintain stemness in luminal breast cancer cells.	[50]
19.	Sp1	Glioblastoma	It acted as critical transcriptional factor to maintain stemness in GBM cells	[51]
20.	Specific small ubiquitin-like modifier (SUMO) proteases 1 (SENP1)	Hepatocellular carcinoma	It enhanced the desumoylation of HIF-1α to promotes the stemness in HCC cells	[52]
21.	IL33	Colon Cancer	It expressed in vascular endothelial cells of colon cancer TME and induced JNK phosphorylation to promote expression of stem cell genes.	[53]
22.	IL-6	Osteosarcoma	It derived from mesenchymal stromal cells and promotes the osteosarcoma Stemness	[54]
23.	MUC1	Lung Cancer	It promoted the tumor-associated macrophages-mediated lung cancer stemness	[55]
24.	BCLXL	Colorectal Cancer	It derived from human colonic fibroblasts and regulated stemness in colon cancer cells	[56]
25.	ILs-3, 6 and 11	Prostate cancer	It promoted the stemness markers SOX2, CD44 and ABCG2 expression in prostate cancer cell lines	[57]
26.	Laminins	Glioma	It promoted the stemness in 3D model of glioma cells	[58]
27.	Microsomal PGE synthase-1 (mPGES-1)	Prostate cancer	mPGES-1 expressed prostate cancer cells showed stem-cell-like features to promote the CD44, Nanog and Oct4 levels.	[59]
28.	Glioma-associated-human MSCs	Glioma	It derived from stromal component of gliomas to increase the self-renewal potential in glioma cells	[60]
29.	Endothelial cells Endothelial Jagged1	Breast cancer	It promoted the enrichment of CD44 cells population and specifically endothelial Jagged1 enhance the notch signaling in breast cancer cell lines	[61]
30.	Interleukin-22 (IL-22) CD4(+) T cells	Colorectal cancer	It activated the STAT3 and histone 3 lysine 79 (H3K79) methyltransferases (DOT1L) and induced stemness via NANOG, SOX2 and Pou5F1 in colorectal cancer	[62]
31.	α6β1 integrin	Breast cancer	It was expressed in CD44 (high)/CD24(low) epithelial and mesenchymal population and controlled by VEGF signaling determine the breast cancer stem cell fate.	[63]
32.	IGF2	NSCLC	It derived from CAFs and induced the Nanog expression to promote stemness in NSCLC.	[17]
33.	Myeloid cells	Pancreatic cancer	It activated STAT3 and increase the ALDH1 positive population.	[64]
34.	CD44	Melanoma	It expressed in CAFs on hypoxic and hypo nutritional conditions in melanoma cells	[65]
35.	High-density mammary fibroblasts	Breast cancer	It involved JNK1 and TGF-β signaling to promotes stemness	[66]
36.	TGF-β	Gastric Cancer	It derived from CAFs and regulate the stemness in gastric cancer cells.	[67]
37.	Myeloid-derived suppressor cells (MDSCs)	Ovarian carcinoma	It targeted the miRNA101-CtBP2 axis to enhance stemness gene expressions.	[68]

#### Immune/Inflammatory cells mediated stemness maintenance

##### Inflammatory cytokines/cells

Interferons (IFNs) are cytokines that regulate various biological activities such as immune response, cell proliferation, and viral infections. It is classified into three major types. Among type1 (IFN α; IFN β) and type II (IFN γ), and type 3 are all presented in mammals [69]. The innate and adoptive immune cells, such as cytotoxic CD8 + T lymphocytes (CTLs) and natural killer (NK) cells in the TME, produce IFNs [70]. A growing body of evidence from the literature suggests that IFNs play a key role in CSC maintenance and cancer cell proliferation, metastasis, and therapy resistance to stimulate CSC makers’ expression to enhance the metastatic potential in pancreatic cancer cells [71]. Specifically, IFN‑γ regulates the chronic myeloid leukemia stem cells to induce proliferation and differentiation [72]. Yamashina et al. [73] showed that drug-resistant tumors containing cancer stem‑like cells produce IFN‑regulated transcription factors, which promote a stem cell phenotype and the production of macrophage colony‑stimulating factor (M‑CSF). Another group reported that JAK signaling regulates stemness through IFN‑γ in bladder cancer cells [74]. Li et al. [75] showed that CD133 + liver CSC populations were enriched by IFN‑γ treatment in hepatocellular carcinoma. Type I IFN maintains stemness in colonic epithelial stem cells. Recently Minamide et al. [76] showed that the organoid-forming ability was significantly decreased in colonic epithelial stem cells of conditional knockdown Irf2^ΔIEC^ colitis mice model. Low levels of IFN γ-induced stemness in a dose-dependent manner to activate the ICAM-PI3K-Akt-Notch1 axis in non-small cell lung cancer [77]. Also, another study showed that basic transcription factor 3 (BTF3), a transcription initiation factor in RNA pol II promoted the stemness and suppressed the interferon regulatory factor 7 (IRF7) in triple-negative breast cancer [78].

Tumor necrosis factor α (TNF-α), a pleiotropic cytokine, is one of the major elements regulating the TME and plays a vital role in inflammation, immunity, apoptosis, angiogenesis, stemness, and cancer progression [79]. It contains two family members, including TNF‑α and TNF‑β. TNF‑α accounts for 70–95% of biological activities and is based on the cytolysis of certain tumor cell lines, and was used as a potential anti-cancer therapy [80]. TNF‑α promotes stemness by inducing the EMT pathways, and it was well demonstrated in renal and cholangiocarcinoma cancers [81, 82]. TNF‑α promoted the sphere-forming ability, stem cell marker expressions, and tumorigenesis in oral squamous cell carcinoma cells [83]. TNF-α plays a vital role in up-regulating EMT marker SLUG to regulate NFκB/HIF1α signaling and maintain the stem-like cell features [84], suggesting that the TNF‑α treatment increased the stem-like cells. Colon cancer cells were enriched in stemness through atonal homolog 1(ATOH1) protein by stabilization of TNF‑α [85]. TNF‑α was secreted in myeloid leukemia CSCs to promote the NFκB p65 pathway and assist CSC survival [86], and protect the colorectal CSCs [87]. Liu et al. [88] found that TNF‑α promoted a transcriptional co-activator TAZ expression to initiate the self-renewal capacity of human breast cancer cells through NF-κB pathway. Chai et al. showed that suppression of this enzyme reduced the stemness in KRAS-driven pancreatic and breast cancer cells [89]. Another group demonstrated TNF’s role in stemness by showing that high-risk human papillomaviruses promote oral carcinogenesis in HPV16-immortalized cells via TNF-α-mediated stemness enrichment [90].

Interleukin 6 (IL-6) is released by macrophages, T cells, and fibroblasts in the TME and maintains a pro-tumor environment on other cells in the TME to encourage tumor evasion and angiogenesis. It interacts with cancer cells via various downstream mediators to promote cancer growth, and their signaling has been shown in various mice and human models [91]. IL-6 plays an imperative role in the stemness enrichment of cancer cells. It promoted self-renewal properties of the CD133^+^ positive population in lung cancer cells [92]. IL-6 induces DNA repair in CD133^+^ CSC-like cells in response to the radiation treatment of lung cancers [93] and promotes stemness during the cisplatin resistance in lung cancer cells [94]. IL-6 regulates the JAK2/STAT3 axis to up-regulate DNA methyltransferase 1(DNMT1), enhancing stem cell proliferation in lung cancer [95]. Ovarian mesenchymal stem cells (OvMSCs) secrete IL-6 and SKOV3 cells exposed to this conditioned media, resulting in enhanced tumor sphere formation and activation of STAT3. Further, OvMSCs with SKOV3 cells in NOD-SCID mice enhanced tumorigenesis [96]. Colonic myofibroblasts are CD90^+^ innate immune cells secreting IL-6, which favors stemness and enhances adaptive inflammatory response to assist tumor growth in colorectal cancer [97]. IL-6 modulates the stemness in osteosarcoma and promotes SOX2, OCT3/4, and NANOG protein in a time-dependent fashion. In sarcoma cells, knocking down IL-6 causes a decrease in self-renewal ability and that of CD133+/CD44 + populations via the osteopontin (OPN)-STAT3 axis [98].

Targeting IL-6 as a therapeutic approach to overcome chemoresistance in tumor formation is critical, and HIF is required for cancer cell chemoresistance. IL-6 increased the chemoresistance of ovarian cancer cells in vivo and in vitro, according to a recent study, by upregulating HIF-1 and STAT3 signaling [99]. BMI1 is a master stem cell regulator responsible for chemoresistance in head and neck squamous cell carcinoma cells. Herzog et al. [100] found that blocking the IL-6 receptor with tocilizumab, a humanized monoclonal antibody, reduced BMI1 expression, sphere formation, and CSC intrinsic chemoresistance significantly. Further IL-6 receptor blockade abrogated the cisplatin-mediated CSC fraction increased and suppressed cisplatin-resistant head and neck squamous cell carcinoma cells xenograft growth.

IL17 is another important cytokine derived from TME. IL-17 is secreted as a disulfide-linked homo and hetero dimer peptide with six members (IL-17 A, IL-17B, IL-17 C, IL-17D, and IL-17E) [101]. Yang et al. [102] showed that colorectal cancer-derived Foxp3^+^IL-17^+^ T cells possess the ability to promote tumor-initiating cells. This research reveals that co-culturing spherical cells with Foxp3 + IL-17 + T cells dramatically increased the stemness markers CD133, CD44s, CD166, EpCAM, and ALDH1. Further, they validated this effect using a neutralizing anti‑IL‑17 antibody and observed that the stemness effect was abolished after the antibody treatment. Further, Xiang et al. [103] showed that IL-17 promoted the self-renewal potential of ovarian CD133^+^ cancer stem-like cells. Ovarian CD133^+^ cancer stem-like cells significantly increased sphere formation ability by IL-17 overexpression, which might be mediated by NFκB and p38/MAPK signaling pathway. Another study showed that IL-17E binding to IL-17RB activates the JAK/STAT3 and NFκB pathways to promote stemness in human hepatocellular carcinoma cells. This effect is revoked by using JAK and NFκB inhibitors [104]. IL17 and IL17R interactions promote stemness due to the auto/paracrine cytokine feedback loop [105]. Further, Jiang et al. [106] showed that IL-17 positively influenced the transformation of quiescent gastric CSCs into invasive phenotype and further confirmed that this effect was mediated by the STAT3 pathway. The mucin glycoproteins enhanced the therapy resistance to promote stemness in pancreatic cancer. Interleukin-17 receptor B promoted MUC1 and MUC4 expression, enhancing stem-like properties and resistance to gemcitabine treatment in pancreatic cancer cells [107]. Further, IL-17 facilitated the development of cisplatin resistance in colorectal cancer cells by inhibiting cell apoptosis [108]. These studies suggest that IFNs, TNF-α, IL-6, and IL17 play a pivotal role in enrichment stemness from TME-released factors. Therefore, the blockade of IFNs, TNF-α, IL-6, and IL17 may be a useful therapeutic approach to modulate the severity burden of cancer patients.

##### Myeloid-derived suppressor cells (MDSCs)

MDSCs are vital components in the TME, promoting cancer growth, stemness, and metastasis. MDSCs recognize other immune cells to produce cytokines and execute immunosuppressive functions. MDSCs act as a major immune response regulator and a key target for cancer treatment [109]. A study advocated that a cross-talk between MDSCs and CSCs drives cancer progression, such as CD44^+^ stem-like cells promote regulatory T cells and inhibit T cell proliferation compared to CD44 stem cells [110]. Further, glioblastoma stem cells produced high macrophage migration inhibitory factor (MIF), which increases the immuno‑suppressive enzyme Arg1 in MDSCs dependent on the CXCR2 axis [111]. MDSCs efficiently utilize miRNAs to enrich stemness in ovarian carcinoma cells. MDSCs triggered the miRNA101 in cancer cells and regulated the co-repressor gene C‑terminal binding protein‑2 (CtBP2) to induce stem cell properties [68]. Another study revealed that the monocytes were transformed into monocytic MDSCs in pancreatic TME to enhance stemness and CSC mesenchymal properties [64]. Ai et al. (2019) showed that MDSCs maintain stemness by promoting piRNA-823 and DNMT3B activation in multiple myeloma cells and provide a novel target for MDSC and CSCs in multiple myeloma microenvironments [112]. Recently, granulocyte-MDSCs promoted colorectal cancer stemness in mice through exosomes. They showed that S100A9 was a highly expressed molecule in the exosomes [113]. Overall, these studies confirmed that MDSCs promote the CSC population and could be viable targets for cancer treatment, but still need the effort to identify novel mechanisms in TME-mediated stemness in all types of cancers.

##### Tumor-associated macrophages (TAMs)

IFN-(lipopolysaccharide) classically activates M1 macrophages, causing them to produce cytokines and initiate the immune response. M2 macrophages are alternatively activated by cytokines IL-4, IL-10, and IL-13 to produce the polyamine or proline to induce proliferation and collagen production in wound healing [114]. These M2 macrophages, known as tumor-infiltrating cells in most solid malignancies, play a crucial role in proliferation, invasion, migration, and metastasis [115]. These macrophages evolve as tumor-associated macrophages (TAM) based on tumor cell-secreted chemokines, cytokines, other growth factors, and conditions associated with tumors such as inflammation, lactic acid, anoxia, and blood-containing monocytic series [116]. TAMs provide a unique niche for CSC maintenance and promote CSC survival, migratory potential, and cross-talk between these two cells, which may help understand the role of molecular mediators in cancer pathogenesis. Mounting works of literature support the relationship between the TAM and CSC. Their interaction plays a crucial role in cancer progression, invasion, metastasis, and therapy resistance in the tumor microenvironment [117]. Milk-fat globule-epidermal growth factor-VIII (MFG-E8), a downstream factor for TAM, activates the STAT3 and sonic Hedgehog pathways to regulate the CSCs [118]. Breast cancer cell line MCF-7 co-culture with M2 macrophages promotes invasive phenotype, demonstrating the TAM’s role in fostering stemness in breast cancer [119]. TAMs facilitate the invasion of glioma stem‑like cells to induce the EMT and production of MMP9 and TGF‑β1 to drive tumor progression [120] and promote TGF‑β1/EMT pathways in hepatocellular carcinoma [121]. Yang et al. [41] showed that IL-10 derived from M2 macrophage promotes the stemness in non-small cell lung cancer cells via JAK1/STAT1/NF-κB/Notch1 signaling, and further, they confirmed that blockage of this pathway could inhibit tumor growth in an *in vivo model*. Further, lung cancer cells co-cultured with M2 TAMs significantly increased mucin protein MUC1 and stemness gene expression. MUC1-silenced M2-TAMs showed fewer stemness features and concluded that TAMs play an essential role in lung cancer progression [55]. Huang et al. [122] found that TAM-derived CCL5 promotes prostate cancer cell self-renewal and that knocking CCL5 out of TAMs suppresses xenograft growth, bone metastasis, and self-renewal properties of these cells, confirming their role in β-catenin/STAT3 signaling. The above-mentioned studies consolidated the role of the TME component, i.e., TAMs’ role in CSC maintenance and cancer progression.

#### Stromal cell-mediated stemness maintenance

##### Fibroblasts

**Fig. 4:**
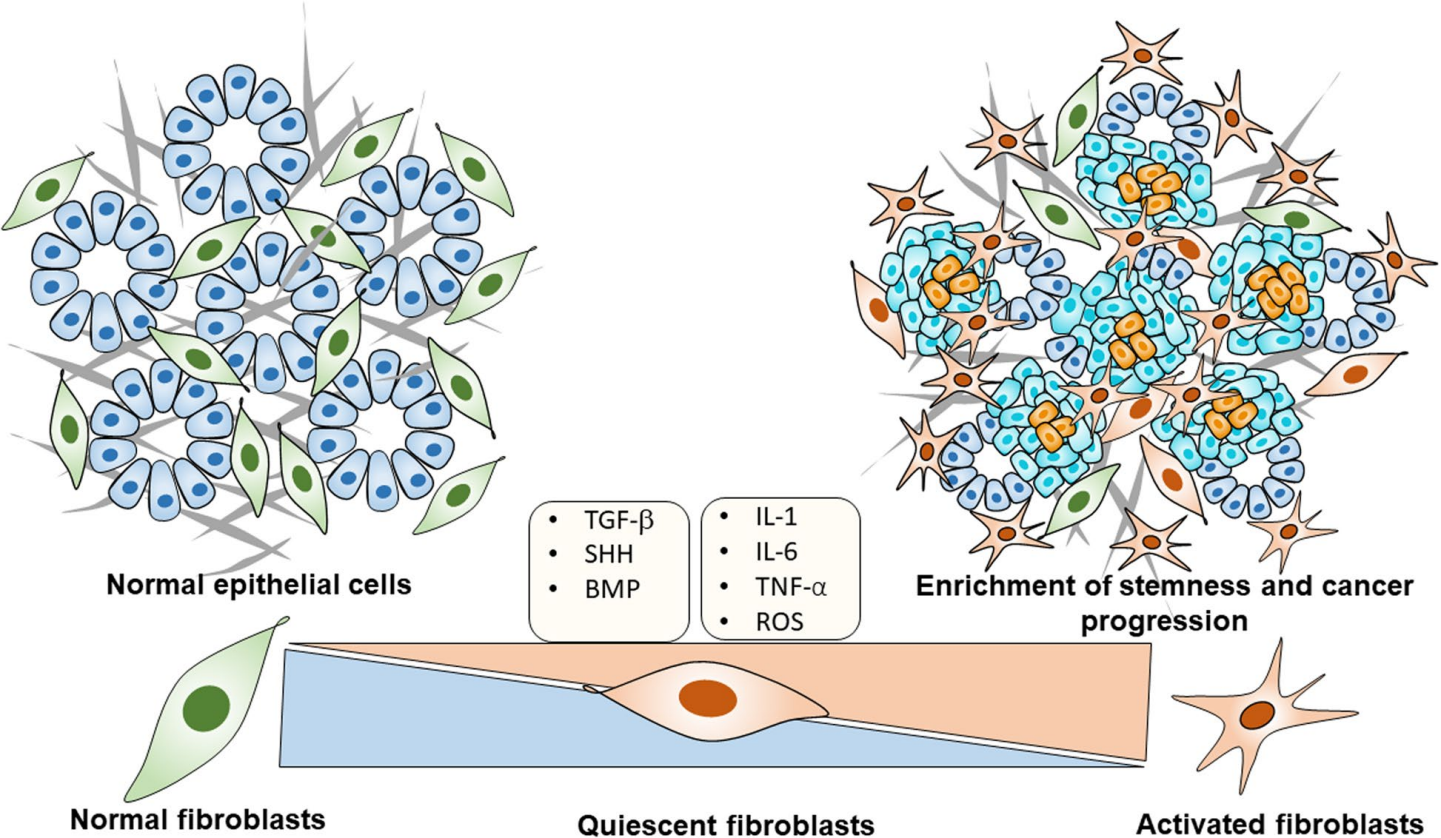
The proposed mechanism for activating the microenvironmental tumor component, fibroblast activation, to enrich stemness in cancer cells: A normal healthy pancreas (denoted blue color) cells surrounded by a normal fibroblast (denoted green color) provided the mechanical and physiological functional support to the pancreas; however, transition into pancreatic tumor cells (denoted sky blue) released cues (TGF-β, IL-6 etc.) and other external factors (ROS) surrounding the tumors made quiescent fibroblasts converted into activated fibroblasts (denoted red color). This vital activation mechanism is considered a crucial event in the enrichment of stemness in tumors (For interpretation of color references in this figure, the reader may suggest referring to the web version of this article). Abbreviations: TGF-β-Transforming growth factor-β; SHH- Sonic hedgehog; BMP4- Bone morphogenetic protein 4; IL6-Interleukin 6; TNF-α-Tumor necrosis factor-α; ROS-reactive oxygen species

Fibroblasts are a common stromal cell type found in the connective tissue and produce the extracellular matrix and collagen to maintain a structural framework of the tissues. It has a branched cytoplasm containing a speckled nucleus with two or more nucleoli. Fibroblasts secrete precursors of ECM to support a structural framework on connective tissue and play a significant role in the wound healing process [123]. In normal physiological conditions, fibroblasts play a vital role in tissue repair to coordinate highly organized. In cancerous conditions, fibroblasts become activated due to various stimuli and get transformed to acquire immune-suppressive, pro-inflammatory, and tumor-promoting phenotypes called “cancer-associated fibroblasts (CAFs).” We described the proposed mechanism of activation of fibroblast in Fig. 4. CAFs originate from progenitor cells such as tissue fibroblasts, endothelial, epithelial cells, bone marrow-derived mesenchymal stem cells, and hematopoietic stem cells [124]. Further, the resident fibroblast converted into trans-differentiated myofibroblasts received a series of cues such as platelet-derived growth factor (PDGF), basic fibroblast growth factor (bFGF), and TGF growth factors [125].

The trans-differentiated myofibroblasts further received cues from different sources to activate and accrue heterogeneity to maintain the expression of several markers [125]. These activated CAFs are spindle shapes associated with large nucleoli and myofilaments and express a broad range of markers such as α-SMA, FAP, FSP, S100A4, PDGFR α/β, tenascin-C; however, none of the markers are expressed specifically [126]. Studies have consistently demonstrated that CAFs dominate tumor progression in all solid malignancies [127]. CAF-driven tumorigenesis in human cancers suggests that activated fibroblasts are likely to play a key role in tumor formation, which needs further investigation.

##### CAFs and stemness

CAFs are a major source of extracellular matrix synthesis in the tumor stroma. They deliver a wide range of soluble factors that aid and maintain tumor progression, angiogenesis, and the CSC niche and promote drug resistance [128]. Giannoni et al. [129] first showed the functional role of CAFs in prostate cancer cell stemness. They identified that IL-6 was secreted from prostate cancer cells to activate the CAFs to release the MMPs. These MMPs promote EMT and enhance the stemness markers and self-renewal prostaspheres. This study also investigated the paracrine signaling between CAFs and prostate cancer cells to demonstrate the stemness properties linked to prostate cancer metastatic aggressiveness. Further, they proved this effect is mediated by the COX-2/HIF-1/NF-κB signaling mechanism [130]. CAFs promoted the reverse Warburg effect, leading to oxidative stress in the TME and providing lactate and ketone bodies to generate ATPs to the progression of tumor growth [131] and promoted stemness [132]. Further, Kinugasa et al. [65] showed that CAFs maintained stemness in vivo and in vitro models. This study revealed that stemness marker CD44 was highly expressed in CAFs and confirmed that CD44 contributes to the maintenance of CSCs in TME and is involved in the therapy resistance in CAFs. CSCs isolated from cancer cells could not maintain their stemness for an extended period; whether indirect interaction with TME or TME feeder cells, CSCs must maintain their stemness. Co-culturing CD90-positive CAFs with OCT3/4, Nanog expressed lung CSCs resulted in stem cell-like properties. They also discovered IGF1R activation and IGF-2-induced Nanog expression and stemness in non-small cell lung cancer CAFs [17]. Scirrhous gastric cancer CSC and OCUM-12/SP cells were exposed to CAF-condition media significantly increased stemness markers, and further, these effects have declined the use of TGF-β inhibitor. This study concluded that CAFs promoted stemness in gastric cancer cells through TGF-β signaling [67]. CAFs released a factor HMGB1 to activate the TLR4 to maintain the stemness in breast cancer [50]. CAFs secreted IL-6 promoted the STAT3/Notch signaling to maintain the stem-like status [133]. The secreted hepatocyte growth factor (HGF) regulates the c-Met/FRA1/HEY1 axis in hepatocellular carcinoma [134]. In 2017, Nair et al. showed that CAFs activation was the source of CSC enrichment. They developed CSC-like cells from mouse-induced pluripotent stem cells exposed to condition media collected from breast cancer cells. They observed that CSC-like cells were surrounded by myofibroblast-like cells. The CSC-derived CAF maintained the tumor microenvironment survival and showed the expression of specific FSP, FAP, and α-SMA markers [135]. CD10^+^/GPR77^+^ CAFs have been identified to be a subtype of CAFs found in breast cancer patients to promote stemness and drug resistance by cleaving C5a, a protein fragment produced by complement component cleavage [19]. Further, CAFs endorsed stemness was studied well in breast cancer [136], ovarian cancer [137], and lung cancer [19]. We recently identified that CAF promoted the enrichment of the stemness population through osteopontin (OPN) or secreted phosphoprotein 1 (SPP1) and CD44-mediated cellular cross-talk in pancreatic cancer cells. We also showed that stemness marker CD44 and stromal marker α-SMA were positively correlated and progressively increased from the early to late stages of KPC mouse and human pancreatic tumor specimens [138]. CAFs also regulated immune cells and stemness in cancer cells through a 5-lipoxygenase-mediated mechanism by condition media collected from CAFs incubated with myeloid-derived suppressor cells promoted stemness and chemoresistance in intrahepatic cholangiocarcinoma cells [139]. So, CAFs fostered stemness features and reprogrammed therapy resistance and metastatic cascade events.

##### Endothelial cell -mediated stemness maintenance

Endothelial cells are specialized cells forming the linings of blood vessels, creating a life-supporting system for migrating cells in every part of the body. Further, endothelial cells can remodel and extend the blood vessel network and tissue growth. Endothelial cells containing the capillaries related to arteries and veins in the vascular system create the vascular bed of the whole body. This vascular system is required for normal physiological functions such as organ development and tissue regeneration. So, tumor vasculature was established through a critical process known as neo-angiogenesis for tumor survival and expansion. RNA sequence experiments elucidated the distinct lineages of endothelial cells in TME. These subgroups of endothelial cells promoted the cancer progression from the non-invasive to the invasive stage of tumor development [140]. Further endothelial cells secreted endocrine factors, critical players in tumor development [141]. Tumor endothelium was thought to be a potential target for cancer therapy. However, clinical trials for tumor angiogenesis-based targeted therapies failed because these endothelial cells cross-talk with CSC, which play a large role in tumor progression and therapy resistance.

Lu et al. [142] showed that liver parenchymal endothelial cells (LPECs) secreted factors that promoted the CSC phenotype in colorectal cancer cells. Further in that study, they showed that CD133 positively stained colorectal cancer cells located in proximity to CD31 positive endothelial cells in human colon cancer tissues. Additionally, they confirmed that endothelial cell condition media-derived jagged-1 via a disintegrin and metalloproteinase 17(ADAM17) proteolytic activity leads to activation of NOTCH signaling in the maintenance of stemness of colorectal cancer cells. A study showed that endothelial cells co-cultured with breast cancer cells enriched the CD44 high CD24 low stem cell population in breast cancer cells [61]. Tumor microvascular endothelial cells condition media promoted the CSC phenotype in differentiated glioblastoma cells. Further demonstrated that fibroblast-derived growth factor-β secreted from microvascular endothelial condition media is responsible for inducing stemness in those cells [143]. Wang et al. [144] showed that condition media collected from endothelial cells isolated from non-malignant liver, lungs, colon mucosa, and kidney promoted the CSC phenotype in colorectal cancer cells. In that study, they showed that all organ endothelial cells’ condition media increased sphere-forming ability and protein expression of NANOG, OCT4, and 5-fluorouracil chemoresistance in colorectal cancer cells. They also elucidated that Nanog homeobox pseudogene 8(NANOGP8) is highly expressed using enzyme digestion and luciferase reporter assays. Finally, they showed that this effect was mediated by activating the AKT pathway in colorectal cancer cells. Recently McCoy et al. [145] showed that endothelial cells promoted cancer stem cell properties through IL-8 mediated pathway. Further they co-cultured glioblastoma spheroids with brain endothelial cells in microfabricated collagen gel to mimic 3D in that study. They identified that endothelial cells increased CSC enrichment via IL-8. Wang et al. [146] showed that endothelial cells activated the epidermal growth factor receptor 3 (HER3)-AKT pathway in paracrine to promote colorectal cancer survival. This research revealed that condition media derived from liver parenchymal endothelial cells (LPECs) increased colorectal cancer cell proliferation and chemoresistance by activating AKT. This was found using a tyrosine kinase receptor array, which revealed that HER3 was significantly elevated in LPECs condition media. Qiao et al. [147] co-cultured human umbilical vein endothelial cells with human monocyte cell line (THP1) using sodium alginate bead-based 3D model, significantly increased CSC features and MDR1 expression in THP1 cells. According to the findings studies, tumor microenvironmental endothelial cell cues play a critical role in cancer cell stemness and therapy resistance.

##### Adipocyte-mediated stemness maintenance

Adipocytes are specialized energy-storing fat cells primarily composed of adipose tissue in the whole body. Adipocytes are white and brown adipocytes in which white adipocytes secrete hormones, adipokines such as resistin, adiponectin, leptin, and brown adipocytes provide to generate heat for the body [148]. Normal adipocytes that were stimulated by cancer cells became cancer-supporting or cancer-associated adipocytes. Cancer-associated adipocytes secreted adipokines, cytokines, and growth factors that inhibited endocrine signaling to tumors and-induced stemness enrichment [149]. A recent study showed that adipose-derived stromal cells expanded the CD44 v6 positive metastatic colorectal cancer cell compartment, which secreted neurotrophins, nerve growth factor, and neurotrophin-3 to recruit the adipose stem cell population within the tumor mass. Further, these visceral adipose-derived factors promoted vasculogenesis and metastatic dissemination by activating the STAT3 pathway, which inhibited miR-200a and enhanced ZEB2 expression in colorectal cancer cells [150].

Several studies have shown that adipose-derived stem cells play a role in cancer progression and enrich the stemness in cancer cells. Multipotent adipose-derived stem cells can differentiate into osteocytes, myocytes, and neuron-like cells. Adipose-derived stem cells are characterized based on their CD13, CD31, CD34, CD45, CD73, CD90, and CD105 markers expressions on their surface [151]. Goto et al. [152] demonstrated that adipsin, an adipokine secreted from mammary adipose tissue, promotes tumor growth and stem cell-like properties in human breast cancer patient-derived xenograft tumor cells. Furthermore, in that study, adipose-derived stem cells significantly enhanced the sphere-forming ability of patient-derived xenograft cells. Furthermore, they found that blocking specific adipsin inhibitors reduced sphere-forming ability and CSC marker expression in co-cultured breast cancer patient-derived xenograft cells. This study concluded that adipsin secreted by mammary adipose tissue functions as a functional component of the tumor microenvironment and CSC niche development in breast cancer.

Adipose-derived stem cells in the TME could influence the therapeutic sensitivity of various cancer. Condition media collected from human adipose-derived stem cells promoted doxorubicin resistance in triple-negative breast cancer cells [153]. Adipose-derived stem cells fused with breast cancer cells showed the enrichment of CD44 + CD24-/low EpCAM^+^ CSC marker expression in the fusion cell population. Interestingly, they found fusion efficiency was high in CD44-enriched MDA-MB-231 breast cancer cells with adipose-derived stem cells fused hybrid cells. The knockdown of CD44 significantly reduced cell fusion efficiency. According to the findings of this study, CD44-mediated cell fusion is critical in developing CSC in breast cancer cells [154]. Adipose-derived stem cell secretome promoted the proliferation apoptosis and increased CD44^+^ CD24^−^ tumor-initiating cells in MCF-7 and MDA-MB-231 cells [155]. A recent study developed adipose-derived stem cell secretome dose-dependent inhibition of triple-negative breast cancer. The efficacy of secretome formulation with monotherapy and combination therapy with paclitaxel was investigated. The secretome formulation significantly decreased CD44^+^/CD24^−^, MDR1^+^ and PDL1^+^ populations in triple-negative breast cancer cells and declined the in vivo tumor growth. When combined with other standard treatments, secretome formulation was found to be a viable biotherapeutic option for breast cancer patients [156]. It was concluded that secretome formulation is a potential biotherapeutic option for breast cancer patients when used with other standard care therapy. Overall, these studies concluded that cancer-associated adipocytes and adipose-derived stem cells are potential players in the TME-promoted stemness in various cancers.

### TME and CSCs’ role in therapy resistance

TME plays a vital role in protecting cancer cells from drug action in all solid human malignancies. TME mediates their activity through cross-talk with CSC and other TME cellular components to develop therapy resistance [157]. CAFs, the predominant stromal cell type in the TME, play a pivotal role in therapeutic resistance in many malignancies. Further, CAFs promote therapy resistance to cancer cells by enriching the stemness features and promoting the synthesis of ECM components, creating a physical barrier to chemotherapeutic drug action. Additionally, CAFs secreted soluble factors communicate paracrine manner with other stromal cells to develop therapy resistance. Recent studies showed that consistently expressed ABC transporter, anti-apoptotic molecules for efflux drugs, inhibited the drug’s apoptotic signaling in CSC [158]. The interaction of TME and CSC involved in the therapy resistance signaling pathways is a potential target for novel therapeutic approaches to improve the patient’s therapy response.

#### CAFs and therapy resistance

**Fig. 5:**
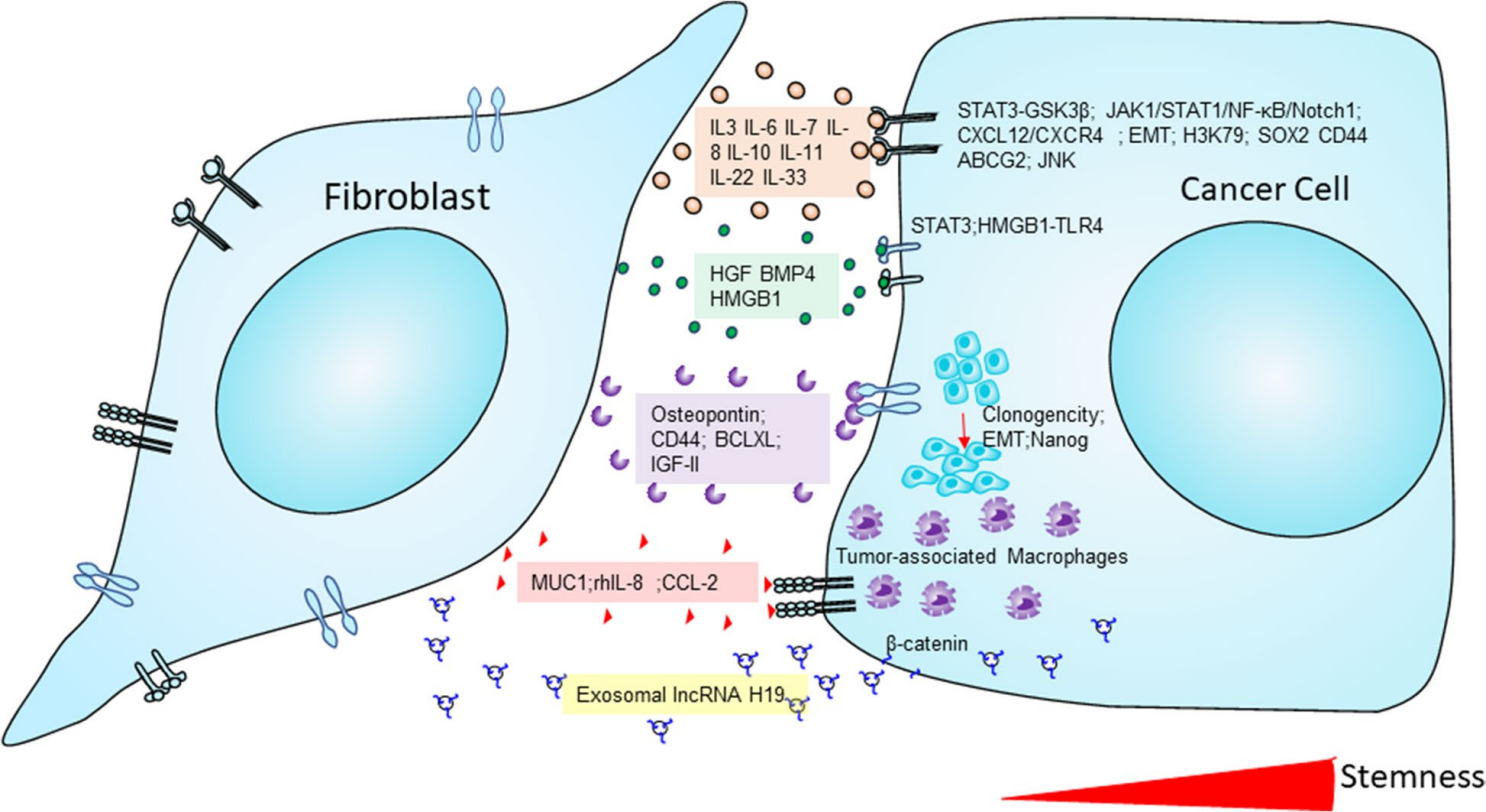
CAFs secretome remodels the cancer stemness maintenance: The diagram depicted the CAFs secretome cross-talk with cancer cells and modulated the enrichment of stemness in the cancer cells. Abbreviations: IL-3,6,7,8,10,11,22,33-Interleukin 3,6,7,8,10,11,22,33; BMP4- Bone morphogenetic protein 4; HMGB1- High mobility group box protein 1; rhIL-8- Recombinant human interleukin-8; CCL2 - CC motif chemokine ligand 2; lncRNA-long noncoding RNAs; EMT- Epithelial-mesenchymal transition

CAFs have a high tumorigenic potential and can further mediate drug resistance and the proliferation of therapy-resistant cells following drug treatment. Additionally, TME plays a crucial role mediate therapy resistance. So, the chemotherapeutic drugs created a non-targeted effect on CAFs in the TME. CAF-released exosomes with long non-coding RNAs improved cisplatin chemoresistance in small lung cancer cells [159]. CAFs-derived exosomes delivered circular RNA circZFR to promote chemoresistance in hepatocellular carcinoma cells [160]. CAFs promoted chemoresistance through Neuropilin 2 (NRP2) in gastric cancer. Further the knockdown of the NRP2 significantly downregulated the 5-fluorouracil resistance in gastric cancer cells [161]. Li et al. [162] found that stressors like irradiation, hypoxia, and chemotherapeutics impacted lung fibroblasts, facilitating chemoresistance in lung adenocarcinoma. In that study, they identified that TNFSF4 was significantly up-regulated in lung fibroblasts. TNFSF4 promoted cisplatin resistance, inhibited apoptosis, and increased the activity of the NF-κB/BCL-XL pathway activity in lung adenocarcinoma cells [162]. In vulvar squamous cell carcinoma, CAFs secreted exosomal long-coding RNA UCA1, promoting chemoresistance via microRNA miR-103a and checkpoint kinase WEE1 G2 [163]. CAFs critically involved in hepatocellular carcinoma chemoresistance by enhancing the CD73 + positive hepatocellular carcinoma cancer cells through the HGF-Met-ERK1/2 pathway. Furthermore, compared to CD73- HCC cells in vivo, CD73-positive cells promoted sorafenib and cisplatin resistance and high tumorigenic potential in CD73 + HCC cells [164]. Targeted chemotherapies modulate CAFs in the stromal microenvironment to promote the development of chemoresistance in cancer patients. Cetuximab monoclonal antibody treatment promoted the secretion of epidermal growth factors in the CAFs to resist cetuximab treatment and increased MAPK signaling in colorectal cancer patients [165]. Wnt Family Member 16(WNT16) is a WNT family member protein closely associated with the development of chemoresistance in cancer cells. Taxotere-treated CAFs were shown to have higher expression of WNT16, and knockdown WNT16-expressed CAFs co-cultured with breast cancer cells significantly increased the sensitivity to taxotere chemotherapy treatment. So, WNT16 expression in CAFs might be contributed to chemoresistance in breast cancer cells [166]. Another study demonstrated that RANBP2-type and C3HC4-type zinc finger-containing 1 (RBCK1) protein was over-expressed chemoresistant colorectal cancer patients. In that study, they showed colorectal cancer cells cultured with condition media collected from CAFs significantly enhanced stemness and chemoresistance with highly expressed RBCK1 protein in colorectal cancer cells. This study indicated that RBCK1 modulated the drug sensitivity of colorectal cancer cells [167]. Lotti et al. (2013) showed that chemotherapy modulated the CAFs activation, and these impacts maintained the stemness in the IL17A-mediated mechanism [168]. In Fig. 5, we elaborated on CAFs secreted factors in inducing stemness potential in cancer cells post introduction of chemotherapy and highlighted the potential role of CAF-CSC cross-talk in onco therapeutic resistance. Overall, TME-mediated CAFs aided cancer stemness by causing pre-metastatic events and therapy resistance and causing the malignancy to interact with other signaling mediators.

#### ATP Binding Cassette (ABC) transporter’s role in therapy resistance

ATP binding cassette (ABC) transporter are the largest family members of transmembrane proteins, with 49 members and seven gene sub families labeled ABCA-G and found in all living organisms. These proteins transport substrate across the intracellular membrane using ATP hydrolysis-mediated energy utilization and have a wide range of physiological functions, including detoxification, oxidative stress defense, xenobiotics, lipid metabolism, antigen presentation, and stem cell drug resistance [169]. These ABC transporters are predominantly expressed in CSCs to correlate with therapy resistance in many solid tumors [170]. Recently Taeb et al. [171] showed that radiation-induced TME component, adipose-derived mesenchymal stem cells (ASCs), impact different cancer cells. They found that radiation-induced ASCs cells condition media exposed cancer cells had high ALDH and ABC transporters expression. Another study showed that CAF-induced ATF4 was highly expressed in PDAC tissue and correlated with poor prognosis. They demonstrated that ATF4 directly binds to the ABCC1 promoter region and regulates the gemcitabine resistance-related gene expressions in pancreatic cancer cells [172]. HIF-2α expression enhanced stemness and adriamycin resistance in ovarian cancer patients. HIF-2α promoted the transcription of BCRP, a second member of the subfamily G of the enormous human ABC transporter responsible for pumping drugs out of the cells to promote drug resistance in ovarian cancer [173]. TME is a crucial regulator of acute myeloid leukemia (AML) blast survival. Mesenchymal stromal cells facilitated ABC transporter with the better survival of blasts in anthracycline-treated AML cells. The stroma-dependent ABC transporter activation leads to the induction of side population phenotype in primary leukemia blasts, and the further transcriptomic study confirmed the enrichment of drug metabolism. This study demonstrated the TME-CSC-induced chemoresistance of AML cells [174].

#### Apoptotic machinery role in therapy resistance

The apoptosis mechanism is activated via two pathways: extrinsic and intrinsic. The extrinsic pathway is initiated by various death receptors (TNF, TRAIL), and the intrinsic pathway is mediated by mitochondria and releases apoptotic factors (cytochrome C). A group of cysteine proteases enzyme “caspases” carried out both pathways [175]. One of the hallmarks of cancer is dysregulated apoptotic machinery to defects in both extrinsic and intrinsic pathways. TME and CSC are major players in the deregulation of apoptotic pathways, according to studies on abnormal apoptotic machinery in many human cancers. Neoplastic cancer cells initiated the inflammatory response in surrounding tissues to modify the environment as a pathological entity. This TME provided appropriate signals for apoptotic resistance to cancer therapies. TME is a pivotal factor in resistance to chemotherapy-mediated apoptosis with their endothelial cells, immune cells, fibroblasts, adipocytes, and mesenchymal stem cells [176].

#### DNA Damage Response (DDR) role in therapy resistance

DNA Damage Response (DDR) is an endogenous cell DNA lesions machinery to repair lesions and maintain genomic stability. TME and CSCs have a capacity-efficient DNA DDR system that protects the cancer cells from DNA-damaging therapeutic agents. Platinum-based therapeutic drugs and radiotherapy eradicated cancer cells using DNA damage. Bao et al. [177] showed that cancer stem cells contributed to the high level of radioresistance in glioma cancer patients to activate the DNA damage checkpoint response and increase DNA repair capacity. Further, they confirmed CD133 positive glioma stem cells were reversed radioresistance using specific DNA checkpoint inhibitors of Chk1 and Chk2. Desai et al. [178] showed CD133^+^ CSC population in lung cancer cells promoted radioresistance through altered regulation of DNA repair genes. Replication stress, a form of DNA damage, is pivotal in therapeutic resistance in CSCs. A recent study showed breast cancer CSCs have less replication stress than non-breast cancer CSCs. The breast cancer CSCs have the BMI1 protein placed around stalled replication forks to engage RAD51 to activate the homologous recombination machinery to confirm that the BMI1/RAD51 axis is necessary to prevent cisplatin-induced cisplatin DNA damage [179]. This study deals with replication response in breast cancer CSCs and provides profound implications for understanding the therapeutic resistance in CSCs.

#### CSC’s survival pathway’s role in therapy resistance

The stemness-related pathways, such as hedgehog, Wnt/β-catenin, and Hippo pathways, were involved in the therapeutic failure of all solid malignancies [180]. Lodestijn et al. [181] showed that PDAC cells have clonogenic potential because they were found close to activated cancer-associated fibroblasts by using maker-free stochastic clonal labeling and quantitative modeling confirmed stroma promoting hedgehog pathway inhibition was altered the tumor growth. This study portrayed the role of TME/CSC-mediated Hedgehog signaling in the development of therapy resistance in PDAC cells. Guo et al. [182] identified that tumor-associated immune microenvironment and hedgehog pathway drive CSC evolution and therapy resistance. They revealed that ubiquitin-specific peptidases, a major family member of deubiquitinating enzymes, have a significant impact on anti-tumor immune response and that inhibiting this protein promoted CSC immune killing ability to reduce chemoresistance in cancer cells. The renin-angiotensin system (RAS), a key regulator of CSC, plays an essential role in TME. RAS components, such as cathepsins B and D, were highly expressed in CSC. Inhibiting these molecules reduced the hedgehog pathway, which could lead to tumor metastasis and therapy resistance in various cancers, according to studies [183].

Colorectal cancer patients are highly suitable for chemotherapy and frequently relapse into the tumor. Stromal factors are responsible for contributing to the chemoresistance of colorectal cancer. Microenvironment components HIF-1α and CAF-derived TGF-β2 promoted the hedgehog transcription factor GLI2 in CSCs, enhancing intrinsic resistance to drug therapy [184]. Human ovarian carcinoma-associated mesenchymal stem cells are multipotent cells that can differentiate into microenvironmental components such as adipocytes, fibroblasts, etc. These cells promoted CSC growth in ovarian cancer and expressed a high level of BMP, a TGF-β family growth factor. Chemoresistance in ovarian cancer cells was reversed using a hedgehog inhibitor or a BMP4-neutralizing antibody. This discovery was linked to other pancreatic and bladder cancers.

Wnt/β-catenin is a highly conserved pathway that regulates cellular events, including proliferation, migration, apoptosis, and stem cell maintenance. This pathway has been identified as an important target for TME-CSC-based therapy resistance. In pancreatic cancer, stromal pancreatic stellate cells secreted Wnt and tenascin C (TnC) ligand molecules promoting the β-catenin and YAP/TAZ signaling pathways. Furthermore, N-Myc downstream-regulated 1(NDRG1) and DNA protein cross-link (DpC) reduced cross-talk between pancreatic cancer cells and pancreatic stellate cells by inhibiting β-catenin and YAP/TAZ nuclear localization and promoting DKK1, a Wnt inhibitor, in pancreatic cancer cells [185]. Mesenchymal stem cells (MSCs) in the TME involved radiotherapy resistance in hepatocellular carcinoma. Hou et al. [186] showed that co-cultured irradiated MSCs and CD133 + hepatocellular carcinoma cells promoted stemness by increased colony formation and wnt expressions in CSCs. In glioblastoma, endothelial cells acquired into mesenchymal stem cells-like cells promoted the therapy resistance. Further, the RNA sequence revealed that the c-Met mediated axis promoted the Wnt signaling, β-catenin phosphorylation at Ser675, and multidrug resistance-associated protein-1(MRP-1) expression leading to stemness and chemoresistance in glioblastoma cells. Wnt inhibitors and temozolomide significantly inhibited tumor growth and extended mouse survival in the glioblastoma CSC model [187].

Hippo pathways play an essential role in TME-CSC-mediated chemoresistance in solid malignancies. Hippo pathways have transcriptional co-activators such as YAP and TAZ that play an essential role in embryonic development, proliferation, cancer progression, CSC maintenance, and therapy resistance [188]. This pathway regulated the cell fate and differentiation of progenitor cells during organ development, and it involved the expansion of dedifferentiated and undifferentiated stem/progenitor cells [189]. A growing body of studies implicated the TME-CSC mediated YAP/TAZ activation in therapy resistance. Tian et al. (2022) showed that YAP/TAZ are key regulators of CSC-related therapy resistance in breast cancer cells. RICH1 protein inhibited the migration and sensitized the chemotherapeutic drugs, and RICH1 activated the kinase pathways of hippo signaling by displacing Amot-p80 protein from a tumor suppressor gene of NF2, named merlin [190]. A large body of evidence suggests that CSC signaling pathways play important roles in therapy resistance and cancer progression. TME-CSC mediated CSC signaling, on the other hand, has only been studied at a preliminary level; more research is still required to reveal the mechanism.

### Role of tumor microenvironment in Cancer stem cells and metastasis

#### Metastasis- the culprit behind cancer patient mortality

Cancer metastasis is a life-threatening event in all solid malignancies, and 90% of cancer patient mortality happens at a later stage of the disease. The failure to manage the disease continually raises cancer-related death each year. Metastasis is a multidimensional evolution of fundamental biological processes in which cancer cells move from their originating site to form a tumor colony in distant organs and tissues. Intravasation, survival, and diffusion into the circulation, extravasation, and the ability of mesenchymal cells to transform into epithelial cells, resulting in colonization and micrometastasis at distant secondary sites [191]. In secondary sites, 1% of disseminated tumor cells can establish macrometastasis. The tumor microenvironment created this inefficiency by regulating the extravasation of a small subset of cells, allowing them to begin growing in additional sites [192]. These small fractions of cells have a strong metastatic potential, relying on wound healing, regeneration, and embryonic development biology to spread to a distant tumor. The ability of these cells to change form during development is critical for immune system survival. As a result, these regenerative cells were re-creating tumors in different places.

Metastasis-initiating cells boost cellular plasticity and stemness by hijacking stem cell pathways, yet, these cells can also survive the metastatic cascade in a specific organ or tissue *milieu*. These cells may reside in original tumors, originate through metastatic cascade events, or develop the ability to engage with microenvironmental components such as immunological, endothelial, and stromal cells after arriving at a distant site [193]. The origin of these cells is unknown, and they are difficult to identify and classify. These metastatic starting cells, according to researchers, exhibit distinct stem-like features and are difficult to identify and analyze using essential research methods.

#### Metastatic Cancer stem cells

In the metastasis beginning site, a tiny subset of metastatic cancer stem cells is capable of invasive and self-renewal capability. Metastatic cancer stem cells have represented the CSC hierarchy with suitable molecular markers in the metastasis region. α-enolase (ENO1), a glycolytic enzyme produced on the surface of tumor cells, has been identified as a key metastasis-promoting factor. ENO1 was recently discovered in the invadopodia surface of the human stomach and prostate adenocarcinomas by Huang et al. [194]. They also discovered that surface ENO1^+^ CSCs with strong pro-invasive and pro-metastatic potential blocked ENO1 expression and downregulated caveolin-1 expression, both of which are necessary for invadopodia formation and CSC invasiveness. In gastric cancer, peritoneal metastatic cancer stem cells (pMCSCs) promote invasiveness and partial EMT in establishing peritoneal tumors. Furthermore, these created pMCSCs consistently expressed CD44 and CD54 markers, which are important in the gastric cancer metastasis. TGF adaptor protein-2 spectrin (SPTBN1) is a critical protein in liver regeneration and acts as a tumor suppressor, and its downregulation has been shown frequently in hepatocellular carcinoma [195]. From an IL-6-treated 2-spectrin (2SP) +/- knockdown mice inflammatory model, CD133 positive stem cells were recovered from the liver. The tumorigenic and metastatic potential of CD133 + liver stem cells is higher than in wild-type control mice treated with IL-6. This study found that inflammatory conditions can change CD133-positive liver stem cells into metastatic stem cells by activating IL-6-mediated inflammatory TAK1-NFκB signaling, which causes hepatocellular carcinoma metastatic development [196].

HIF-1 and HIF-2 were found to govern metastasis-initiating cells and their progenies during hypoxic cancer progression. The tumor survival and invasion of metastasis-initiating cells are influenced by activated hypoxia factors that regulate pluripotency-associated transcription factors, EMT, and angiogenic indicators [197]. The role of CSCs in metastatic cell lines has only been studied in a few research. Chen et al. (2011) discovered the CD44high/CD24low population of the UP-LN1 lymph node metastatic CEA-producing cancer cell line. They also showed that released IFN (and activated NK/LAK) act as a promoter for LAK-resistant CD44high/CD24low cells. This CSCs enhanced the CXCR4^+^ metastatic cancer stem cell population. The unique abilities to switch between cancer stem cells and metastatic stem cells to initiate tumors for appropriate tissue microenvironment instead of host immune surveillance were discovered in this work [198]. So, the surrounding niche plays a key role in the transition and maintenance of metastatic stem cells in the metastasis-initiating site of the tumor.

#### Tumor microenvironmental factors- soil for metastasis-initiating cells

Metastasis-initiating cells have the ability with stem-like and immune-evasive properties. Recent research shows that tumor cell seeding to secondary sites occurs early in the tumor and can be as dormant as single cells or micrometastases. The surrounding tumor microenvironments govern metastatic dormancy and interact with metastatic progeny stem-like cells to trigger colony development in distant sites [199]. Understanding how TME components interact in metastatic cells could be crucial for developing new targeted therapeutics for metastatic diseases.

In the secondary sites, myeloid-derived suppressor cells had a pleiotropic function in shaping the metastatic tumor microenvironment, allowing tumor cells to escape the innate and adaptive immune response [200]. MDSCs cells are collected in putative metastatic sites before cancer cells arrive, producing a “premetastatic microenvironment,“ according to recent research. In breast, bladder, and melanoma malignancies, this niche aids the extravasation of moving cancer cells and facilitates the development of new blood vessels [200]. Further primary tumor-secreted soluble factors also facilitated the development of the pre-metastatic niche that will helps the survival and outgrowth of incoming tumor cells [201]. Tumor-derived substances aided the spread of metastatic colorectal cancer to the liver. Sphingosine-1-phosphate receptor 1 (S1PR1)–STAT3 activation in tumor cells generated IL-6, which activated S1PR1–STAT3 in MDSCs of the liver, resulting in the establishment of a pre-metastatic niche before the entry of colorectal cancer cells, according to a recent study. This research hypothesized that tumor-derived factors are effector molecules in creating niches and promoting organ-specific metastasis [202]. TAMs were important metastasis promoters in the TME, producing growth factors, proteolytic enzymes, and immunological checkpoint proteins in T cells and regulating invasion, vascularization, intravasation, survival, and extravasation in the metastatic cascade events [203].

To cross the endothelium during intravasation and extravasation, cancer cells require the disruption of endothelial junctions, known as transendothelial migration. Tumor cells cause angiogenesis, and new blood vessels weaken the cell-cell connection, allowing them to easily enter the vasculature during the intravasate [204]. VEGF and TGF-β lowered the endothelial barrier to tumor cells entering blood arteries. Cancer cells bind to endothelial cells during extravasation and migrate through the tiny capillary vessel wall by trans-endothelial migration. The endothelium moved into selectins, cadherins, integrins, and CD44, among other ligands and receptors involved in cancer cell adhesion [204]. Endothelial cells form tumors and play a key role in tumor cell metastasis. Biglycan, a proteoglycan released by tumor endothelium cells, stimulated tumor cell migration by activating NFκB and extracellular signal-regulated kinase1/2 [205]. Studies explored the CAFs’ involvement in tumor metastasis. Metastatic fibroblasts formed metastatic lesions, remodeled the ECM of metastatic tumors, stimulated angiogenesis, and formed a pre-metastatic microenvironment [206]. However, further research is necessary to define the critical pathways involved in immunological, stromal, and endothelial components interacting with cells that initiate metastasis. Strengthening anti-cancer immune response and enhancing patient survival will pave the way to target possible pathways/agents employing new therapeutic molecules.

### Therapeutic targeting of TME-mediated CSC maintenance

CSCs were selected based on their surface maker expression to target the therapeutic strategy to eliminate the risk of recurrence in the developmental stage of all human cancers [207]. The therapeutic strategy for CSCs was based on cell type targeting, specific markers, signaling pathways, ABC transporter inhibition, and induced apoptosis. Sonic Hedgehog, Notch, chemokine receptors (CXC1-2, CXCL8), Fak, and Wnt pathways have all been identified as potential clinical target pathways [208]. However, because CSCs and normal stem cells have similar expression, signaling profiles, and regulatory pathways, the therapeutic efficacy, and clinical impact may be less effective. The drugs target CSC markers, surgery, chemo, and radiotherapy and act as a supplement in cancer patients to minimize the recurrence effect [209]. Monoclonal antibody treatment emerged as effective targeted therapy for many human malignancies. A study demonstrated the therapeutic efficacy against target CD44, CD123, and CD47 markers expressed in AML stem cells [210]. FDA-approved drugs were also used to target CSC marker expressions in different cancers. Cetuximab and erlotinib were used to treat CSCs in head and neck squamous cell carcinoma [211]; cetuximab combination with Ixabepilone in triple-negative breast cancer stem cells [212]. ABC transporters were highly expressed in CSCs, which cause drug resistance in the majority of cancer patients. Targeting ABC transporters have been approached to develop four generations of drug treatment to improve drug efficacy. The current treatment uses new technology to improve therapy efficacy, such as applying specific miRNA or nanomedicine for bypassing efflux pumps. However, there is a lack of drugs approved for clinical practice [213]. There has been a tremendous impact in research in recent years to focus on target-based drug treatment. Traditional chemotherapy has a non-discriminatory toxic effect on both normal and cancer cells. The recent discovery of small-molecule tyrosine kinase inhibitors (TKIs) has led to favorable cancer profile drugs that target cancer-specific EGFR inhibitors such as gefitinib and erlotinib lapatinib, afatinib, canertinib, and angiogenesis inhibitors such as sorafenib, sunitinib, and axitinib. These TKIs are substrates for many ABC transporters; however, many are ABC transporter inhibitors [214]. So, the target of the ABC transporter, combined with other therapy strategies, overcomes the drug resistance in CSCs. In cancer conditions, apoptotic machinery was impaired, so reactivation of apoptotic signaling may be a useful approach for minimizing CSCs homing in cancer development. A study observed that JNK inhibitor and TRAIL treatment significantly impact the CSC’s self-renewal potential without impacting tissue-resident CSCs [215]. Finally, some promising therapeutic agents have developed new approaches to target the CSC populations. Studies using retinoic acid and its analogs for differentiation treat stem-like glioma and breast cancer stem cells [216, 217].

TME plays a vital role in regulating stem-like events to maintain the plasticity in cancer cells. So, targeting cross-talk factors between cancer cells and TME may prove powerful approach than targeting CSC alone. CSCs could acquire drug resistance by interacting with TME components. Thus, targeting the TME components may signify an effective therapeutic strategy for TME-mediated stemness enrichment and prevent therapy resistance. CAFs represent a major component of TME and play an imperative role in therapy resistance. Thus, targeting the CAFs may be a critical therapeutic approach to improving clinical outcomes. FAP protein was expressed on the surface of CAF cells. However, recent studies showed that FAP is expressed in bone marrow stem cells. Systemic anti-FAP therapy may be toxic side effects on cells. As a result, nanoparticle-based photoimmunotherapy (nano-PIT) was used to eliminate CAFs in tumor-bearing immunocompetent mice, and this approach also increased T cell infiltration and tumor suppression [218]. Recently B-cell maturation antigen (BCMA)-targeted chimeric antigen receptor (CAR) T-cell therapy was a positive response in multiple myeloma patients. However, the success rate diminished because of the abundance of CAFs in TME. So, CAF surface markers FAP and SLAMF7 inhibition improved the effector functions of CART cells [219].

Immunotherapy approaches to blocking antibodies such as CTLA-4, PD-1, or PD-L1 might benefit advanced cancer patients. Further combination with other checkpoints or therapeutics molecules improved the immune response. PD-1 therapy response was significantly improved using a combination with CSF surface-modified bladder cancer stem cells vaccine [220]. Further CSC-dendritic cell vaccine with CTLA-4 and PD-L1 blockades significantly mitigated the melanoma stem cells in the mouse model [221]. Further ongoing clinical trials in TME and stemness-related are listed in Table 2.

**Table 2:** List of clinical trials ongoing tumor microenvironment and cancer stem cells-related targeted drugs treatment

Drugs	Cancer	NCT No.	Status	References
MK-3475 (Pembrolizumab)	Triple negative breast cancer (TNBC)	NCT02977468	Recruiting	[222]
MK-3475 (Pembrolizumab)	Metastatic melanoma	NCT03534635	Recruiting	[223]
Mifamurtide	Osteosarcoma	NCT03737435	Unknown	[224]
ALKS 4230 Pembrolizumab	Solid Tumor Patients	NCT04592653	Recruiting	[225]
Abemaciclib Nivolumab	Head and Neck Cancer	NCT04169074	Not yet recruiting	[226, 227]
Gadobutrol [F-18] HX4 Gemcitabine	Pancreatic Cancer	NCT01989000	Completed	[228]
Avelumab CTX	Head and Neck Cancer	NCT03844763	Recruiting	[229]
Dacarbazine	Melanoma	NCT04225390	Not yet recruiting	[230]
Plerixafor	Pancreatic Cancer	NCT03954691	Not yet recruiting	[231]
bevacizumab	Breast Cancer	NCT01190345	Completed	[232]
Metformin	Ovarian Cancer	NCT01579812	Completed	[233]
Etoposide Atezolizumab	Small Cell Lung Cancer	NCT05055999	Completed	[234]
Liposomal Doxorubicin Docetaxel Paclitaxel Carboplatin Cisplatin Gemcitabine Topotecan	Ovarian Cancer	NCT03949283	Recruiting	[235]
Carboplatin Irinotecan Etoposide Carmustine (BCNU) Lomustine (CCNU) Temozolomide Procarbazine Vincristine Imatinib	Glioblastoma	NCT03632135	Active, Not yet recruiting	[236]

## Conclusions and future perspectives

CSCs appeared in a heterogeneous population of tumor cells. Therefore, the identification of CSCs remains challenging. Screening, identification, and use of multiple markers that provide specific tumor types and drug responses. Combination therapy may be more efficient than targeting single molecules or pathways. Immunotherapy is a potential option for targeting CSCs with checkpoint molecules; however, CSCs are less immunogenic than non-CSCs and thus have a limited anti-tumor response.

Organoids are an important model for preserving tumor heterogeneity and drug resistance in high-throughput drug screening. Circulating tumor cells are another promising model because they represent genetic and epigenetic characteristics within the tumor and are a popular drug-testing model. Metastatic stem cell interacts with the local and metastatic microenvironment, and updated knowledge about the cellular and molecular mechanisms governing the animal models of metastatic stem cells should shed light on the potential therapeutic application in the TME- mediated CSC role in metastasis.

Transcriptional heterogeneity in TME and cancer cells have undeviating clinical implications. To elucidate the molecular classification of a single biopsy might prevent the unbiased way to imply clinical management; however, single-cell RNA sequence experiments demonstrated the presence of multiple cell populations in various molecular groups within the tumor and elucidated the mutual cross-talk in symbiotic diverse cell populations in the TME.

Targeting CSCs and TME may represent a wise choice; however, the interconnected network and cues are complex and obscure. TME components have different types of cells, cytokines, and growth factors. CSCs have proved to be targeted to improve cancer patients; however, therapeutics of TME-mediated stemness is still challenging. The focus of future clinical trials in TME/CSC may focus on combination therapy to target multiple pathways and careful consideration of reducing the toxicity to normal cells, improving the drug selectivity and efficacy, and exploring the possibility of the mode of delivery. In conclusion, combining TME/CSC targeted therapeutic molecules with conventional chemotherapeutic drugs may provide a better direction for anti-cancer therapy, resulting in improved cancer patient survival rates and reduced recurrence.

## Data Availability

Not applicable.

## Abbreviations

ABC transporters: ATP-binding cassette transporters
EMT: Epithelial-to-mesenchymal transition
ECM: Extracellular matrix
STAT3: Signal transducer and activator of transcription 3
ABCB1: ATP binding cassette subfamily B member 1
MDR1: Multidrug resistance protein 1
CD44: Cluster of differentation 44
PI3K: Phosphoinositide 3-kinase
JAK: Janus kinase
ICAM-1: Intercellular adhesion molecule 1
Notch 1: Neurogenic locus notch homolog protein 1
NF kB: Nuclear factor kappa B
HIF1α: Hypoxia-inducible factor 1-α
YAP: Yes-associated protein 1
TAZ: Transcriptional coactivator with PDZ-binding motif
EpCAM: Epithelial cell adhesion molecule
ALDH1: Aldehyde dehydrogenase 1
MAPK: Mitogen-activated protein kinases
KRAS: Kirsten rat sarcoma virus
MUC: Mucins
CXCR2: C-X-C Motif chemokine receptor 2
miRNAs: microRNAs
DNMT3 β: DNA methyltransferase 3 β
MMP9: Matrix metallopeptidase 9
TGF‑β1: Transforming growth factor β1
α-SMA: α-smooth muscle actin
FAP: Fibroblast activation protein
FSP: Fibroblast-specific protein
PDGR: Platelet-derived growth factor receptor
COX-2: Cyclooxygenase 2
IGF1R: Insulin like growth factor 1 receptor
HMGB1: High-mobility group box 1
TLR4: Toll-like receptor 4
FRA1: Fos-related antigen 1
TNFSF4: Tumor necrosis factor superfamily member 4
UCA1: Urothelial cancer associated 1
HCC: Hepatocellular carcinoma
ADAM17: ADAM metallopeptidase domain 17
PD-1: Programmed death 1
PDL1: Programmed cell death 1 Ligand 1
PDAC: Pancreatic ductal adenocarcinoma
ATF4: Activating transcription factor 4
ABCC1: ATP binding cassette subfamily C member 1
AML: Acute myeloid leukemia
CTLA-4: Cytotoxic T-lymphocyte associated antigen 4
